# Assessment the Level of Comorbid Depression, Quality of Life and Associated Factors Among Patients with Heart Failure: An Outpatient-Based Study

**DOI:** 10.3390/healthcare14030297

**Published:** 2026-01-24

**Authors:** Zekiye Yılmaz, Anmar Al-Taie, İrem Bayol

**Affiliations:** 1 Department of Clinical Pharmacy, Faculty of Pharmacy, Acibadem Mehmet Ali Aydinlar University, Istanbul 34752, Türkiye; zekiye.yilmaz@acibadem.edu.tr (Z.Y.);; 2 Clinical Pharmacy Department, Faculty of Pharmacy, Istinye University, Istanbul 34396, Türkiye

**Keywords:** comorbidity, depression, heart failure, health-related quality of life, Türkiye

## Abstract

**Background**: Heart failure (HF) affects not only the cardiovascular system but also mental health. The majority of patients with HF experience symptoms of mental disorders, such as depression, which are proportionally related to the severity of HF. This results in a significant comorbidity of HF, which might be associated with poor clinical outcomes, including decreased health-related quality of life (HRQOL). In Türkiye, data concerning the extent of this complication among outpatients with HF are limited. Therefore, the aim of this study was to assess the prevalence of depression in outpatients with HF and consequently the HRQOL; the secondary aim was to identify the related factors contributing to the incidence of depression and HRQOL in patients with HF in Bursa, Türkiye. **Methods**: An outpatient, descriptive, observational, cross-sectional study was conducted in a cardiology outpatient clinic in Bursa Province, Türkiye, between September and December 2022. The study was conducted via a validated questionnaire consisting of four sections. Depression was measured using the Beck Depression Inventory (BDI) scale, and the HRQOL of HF patients was evaluated using the Turkish version of the Minnesota Living with HF Questionnaire (MLHFQ). Simple linear regression and multiple linear regression analyses were used to determine the effects of variables. Limitations of the study include its design as a descriptive, observational, cross-sectional study from a single center that relies on self-reported data. **Results**: A total of 166 patients were enrolled, with a mean age of 64.96 ± 11.33 years. Nearly half of the participants had moderate or severe depression (33.1% and 15.7%, respectively). The mean MLHFQ score of the study participants was 54.15 ± 18.20. Patients suffering from severe depression had the lowest HRQOL (71.46 ± 12.4). There was a significant increase in depression level, and a decrease in HRQOL in patients with a duration of HF diagnosis of more than 3 years (*p* = 0.001), a number of HF hospitalizations (*p* = 0.001), and those diagnosed with NYHA class IV (*p* = 0.001). Multiple linear regression analysis revealed a significant relationship between the duration of HF disease, number of comorbidities, number of medications used, and BDI [(0.30 < r: 0.31/0.43/0.43 ≤ 0.70), respectively]. The simple linear regression analysis revealed that the BDI has positive and significant explanatory power for the MLHFQ (F: 168.29; R^2^: 0.51; t: 12.97; *p* < 0.001), and 51% of the change in the MLHFQ score is recorded by the BDI (R^2^: 0.51). **Conclusions**: The results of this study revealed that comorbid depression and HRQOL are closely related. This was observed in nearly half of the patients with HF, who had comorbid moderate and severe depression, which is associated with poor HRQOL. The factors associated with high depression and poor HRQOL were the duration of HF diagnosis of more than 3 years, an increased number of HF hospitalizations, polypharmacy, and NYHA class IV diagnoses.

## 1. Introduction

Heart failure (HF) is one of the most common cardiovascular (CV) disease conditions, with 64 million adults worldwide living with this disease. The exact prevalence is predicted to be relatively high, as studies usually include recognized and/or diagnosed cases [1,2]. It is still considered a public life-threatening condition characterized by significant morbidity and mortality, poor functional capacity, and health-related quality of life (HRQOL) [3]. Moreover, the total number of hospital admissions from HFs is predicted to increase to 50% in the next 25 years due to population growth, aging, and increasing incidence of comorbid diseases [4,5].

HF affects not only the CV system but also mental functions, resulting in complicated outcomes. Previous studies reported that the majority of patients with HF experience symptoms of depression, which is a significant comorbidity of HF [6,7]. The physiological mechanisms underpinning both depression and HF suggest several commonalities. Factors that exacerbate cardiac function in patients with HF are frequently observed in those with depressive disorders. These include a hyperactive hypothalamic–pituitary–adrenal axis, reduced heart rate variability, elevated levels of inflammatory biomarkers, hypercoagulability, engagement in high-risk behaviors, such as smoking and a more sedentary lifestyle, lower adherence to prescribed medication and dietary recommendations, and diminished social support [8,9,10].

The rate of depression is proportionally related to the severity of HF. The prevalence of depression is estimated to be 40–70% in hospitalized patients with NYHA class III–IV symptoms [7,11,12]. Regardless of the patient’s functional status, HF and depression substantially reduce HRQOL, particularly physical functioning and vitality [13,14]. Moreover, despite early initiation of HF management, patients usually suffer from multiple hospitalizations and poor HRQOL [1,15,16]. Earlier studies reported that depression is associated with multiple adverse outcomes in patients with HF, including decreased medication adherence, increased length of hospitalization, poor functional status, and high rates of morbidity and mortality [17,18]. A previous meta-analysis study indicated that severe depression is associated with a high mortality rate [19]. Similarly, another meta-analysis by Sokoreli et al. revealed that HF patients with depression had a 60% greater risk of mortality [11].

Despite these adverse events, depression in patients with HF is still underestimated because of the overlap of symptoms of these two conditions, and depression can easily be misdiagnosed during the routine monitoring of patients with HF [20,21]. For example, tiredness, concentration trouble, weakness, and sleep disturbances may be associated with both HF and depression [22]. In this context, the European Society of Cardiology (ESC) and the American College of Cardiology/American Heart Association (ACC/AHA), and the Heart Failure Society of America (HFSA)guidelines recommend increasing the awareness of depression in patients with HF, while those patients should be routinely screened and treated for any symptoms of depression during hospitalization and follow-up [23,24]. Dekker et al. [12] reported that improved HRQOL is linked to improved symptoms of depression in patients with HF.

Despite the high prevalence and negative impacts of depression, which have increased globally in patients with HF, earlier studies revealed that the psychological elements of patients with HF are not fully investigated and managed in clinical practice for a variety of reasons, including a lack of knowledge about the relationship between psychological alterations and physical results [25,26,27]. A study by Aggelopoulou et al. [26] revealed that patients with HF present severe symptoms of anxiety, depression, and poor QOL. The study concluded that assessing these patients for these psychological symptoms and providing holistic healthcare by a multidisciplinary team can lead to the prevention and early treatment of the disease. Similarly, another study by Mulugeta et al. [27] revealed that more than half of all adults with HF have comorbid depression, which can influence treatment outcomes and QOL; therefore, consideration of multifaceted approaches, such as psychosocial interventions, is needed to reduce the burden of comorbid depression in this population.

On the other hand, considering that previous studies have demonstrated only the prevalence of depression among Turkish people with HF, studies concerning the extent of this issue while assessing depression levels, functional capacity, and HRQOL, as well as the potential factors contributing to their associations in outpatients with HF in Türkiye are lacking. Therefore, the aim of this study was primarily to assess the prevalence of depression in outpatients with HF and consequently the HRQOL; the secondary aim was to identify the related factors contributing to the incidence of depression levels and HRQOL in patients with HF in Bursa, Türkiye.

## 2. Materials and Methods

### 2.1. Study Design

This was a descriptive, observational, cross-sectional study conducted among patients in a cardiology outpatient clinic of a higher specialization training and research hospital in Bursa Province, Türkiye, between 1 September 2022 and 31 December 2022. The study received ethical approval from Acibadem Mehmet Ali Aydinlar University and the Acibadem Healthcare Institutions Medical Research Ethics Committee (2021-15/16).

### 2.2. Study Participants

Patients who were diagnosed with HF on the basis of the New York Heart Association (NYHA) classification [28] and who attended the cardiology outpatient clinic were enrolled in this study. Participants who satisfied the inclusion criteria and agreed to participate in the study were informed about the study’s purpose and description, and written informed consent was obtained from all participants. Furthermore, the participants were assured of their privacy and response secrecy and were told that participation was voluntary. The inclusion criteria were being over 18 years old, being literate, and having a medical diagnosis of HF. Those excluded were patients who had a medical diagnosis of psychiatric disorders, those who were suffering from visual impairment that impeded completion of the self-report survey, those who withdrew from participation, those with missing data, and those who provided incomplete responses to the questionnaire.

### 2.3. Study Sample Size

Convenient sampling was applied for the cohort of HF outpatients, and the sample size was calculated according to the following formula:

(1) $$n={Z^{2}}_{\left(1-\frac{\alpha }{2}\right)}\frac{p(1-p)}{d^{2}}$$

where *n* is the number of participants, *Z*(1 − *a*/2) = 1.96 (confidence interval: 95%), *d* is 0.09, *p* = 48.5%, indicating the prevalence of depression among HF patients [29].

**Figure 1:**
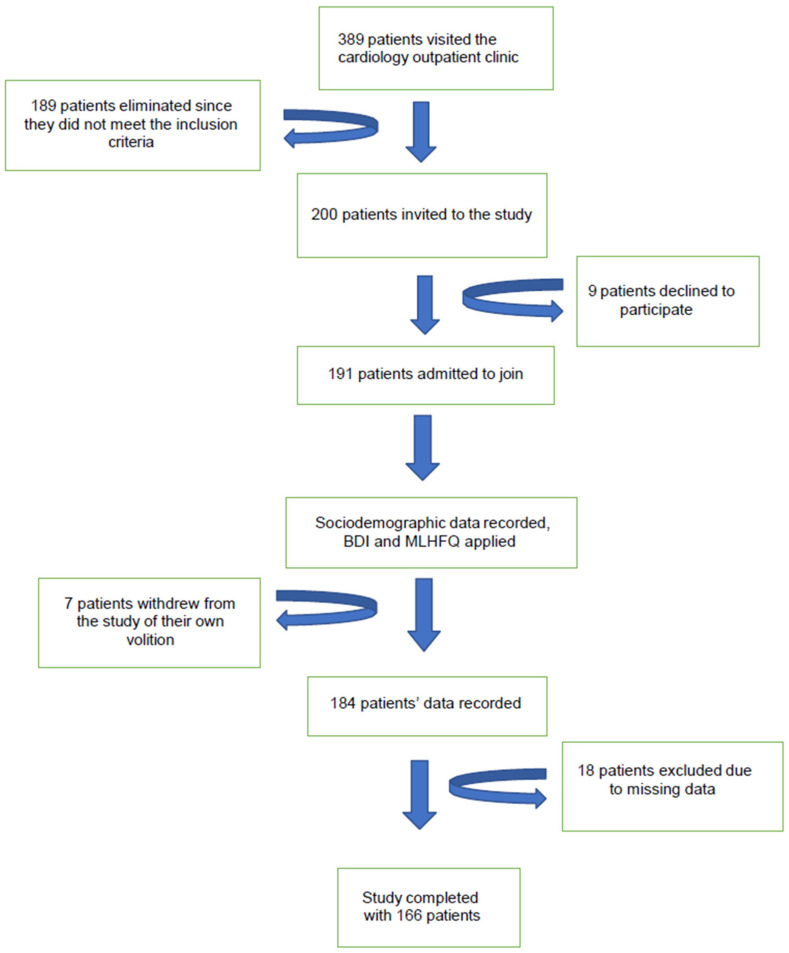
Flow chart of the study participants.

The minimum sample size required was calculated to be 118 participants. The number of patients who met the inclusion criteria and were invited to the study while they were attending the cardiology clinic was 200. However, 9 patients declined to participate, 7 patients withdrew from the study of their own volition, and 18 patients were excluded because of missing data. As a result, a total of 166 patients were enrolled in the study, as shown in Figure 1.

### 2.4. Questionnaire Design and Development

The information was collected using a structured self-administered questionnaire that was developed for the present study. The objectives of the study were described in a face-to-face format. An introductory letter was included within the questionnaire, and filled out by the participants, which took nearly 15 min to complete. The questionnaire consisted of 53 items divided into 3 sections. The first section consisted of eleven items and included patients’ sociodemographic characteristics (age, sex, marital status, education level, working status, cigarette smoking) and disease and medicine usage status (comorbid disease, polypharmacy, which is defined as the regular use of 5 or more medications at the same time, duration of HF disease, hospitalization due to HF, and number of hospitalizations related to HF disease within the last year). The second part included 21 questions to assess depression levels in patients with HF. The third part included 21 questions to assess the impact of HF on patients’ quality of life.

### 2.5. Study Variables and Outcomes

#### 2.5.1. Assessment of Depression Levels in HF Patients

The presence and level of depression in patients with HF were assessed using the Turkish version of the Beck Depression Inventory (BDI) scale. The BDI was developed by Beck et al. [30], and it is one of the most well-researched depression inventories that measures the severity of depression in normal and psychiatric populations. The BDI is not used as a clinical diagnostic instrument but rather as an indicator of the presence and grade of depressive symptoms. The validity and reliability of the Turkish version of the survey were established by Hisli, and permission to use the Turkish version of the survey was obtained from the researcher [31]. The BDI comprises 21 items regarding how the participant feels about several subjects within the last week, and each item is scored on a 4-point Likert scale from 0 points (positive statements about depression) to 3 points (negative statements about depression). Total scores of 0–13 are considered normal, 14–19 indicate mild depression, 20–28 are considered moderate depression, and 29 and over indicate severe depression. The participants can obtain a minimum of (0) and a maximum of (63) points from the survey, and higher total scores represent more severe depressive symptoms [30,31]. Cronbach’s alpha test (α = 0.88 ≥ 0.70) was applied for the inner consistency of the BDI scale used, which sufficiently demonstrated the reliability and validity of the scale used in the study.

#### 2.5.2. Assessment of the Impact of HF on Patients’ Quality of Life

The HRQOL of HF patients was evaluated using the Turkish version of the Minnesota Living with HF Questionnaire (MLHFQ). The MLHFQ was developed by Rector and Cohn to measure the impact of heart failure and heart failure treatment on an individual’s quality of life [32]. The MLHFQ tool has been commonly applied to identify HRQOL among HF patients [33]. The validity and reliability of the Turkish version of the questionnaire were evaluated by Uzunhasanoğlu, and permission to use the Turkish version of the survey was obtained from the researcher [34].

The MLHFQ is one of the most widely used disease-specific health-related quality of life questionnaires for patients with HF. It assesses the patient’s perception of the influence of HF on two dimensions, physical and emotional aspects of life, including mobility, physical symptoms, emotional distress, sleep patterns, social functioning, sexual activity, hospitalization, and medical expenditures. The questionnaire consists of 21 items rated on a six-point scale that assess these various aspects. Each item is scored on a 6-point Likert scale from 0 points (not at all) to 5 points (a lot). The questionnaire is scored by summing all 21 responses and provides a total score that ranges from 0 to 105, with higher scores indicating a poorer quality of life [32,33]. Cronbach’s alpha test (α = 0.93 ≥ 0.70) was applied for the inner consistency of the MLHFQ scale used, which sufficiently demonstrated the reliability and validity of the scale used in the study.

### 2.6. Statistical Analysis

Statistical analyses of the data were performed via the IBM SPSS Statistics 29.0.0 software version. Descriptive statistical analyses, such as frequency, percentage, mean, and standard deviation, were utilized to assess the demographic data. Cronbach’s alpha test was applied for the internal consistency of the scales used. The distribution normality of the study data was examined with skewness and kurtosis coefficients. Variance homogeneity was examined with the Levene test. The mean scores of two independent groups were examined via an independent-samples *t*-test, and the mean scores of 3 or more groups were examined via one-way ANOVA. Simple linear regression and multiple linear regression analyses were used to determine the effects of variables. According to the skewness (−1.5 < SC +1.5) and kurtosis coefficients (−1.5 < KC +1.5), a univariate normal distribution is satisfied for all the variables. According to the Cook distance coefficient (Cookmax < 1), the data are free from extreme values. According to the simple scatter plot, there are linear relationships between the variables. According to the normal P-P plot standardized error graph, the error terms have a normal distribution. According to the Durbin–Watson coefficient (1 < DW < 3), there is no autocorrelation between consecutive observations or error terms. According to the scatter plot, there are linear relationships between the variables, the error terms have constant variance homoscedasticity, and there is no multicollinearity problem between the independent variables and the VIF (VIF < 10) and tolerance (T > 0.10) coefficients. Linear regression analyses were performed based on the least squares method and the enter method. All the data were considered statistically significant at a *p*-value < 0.05 and a 95% confidence interval.

## 3. Results

A total of 166 patients were enrolled, with a mean age of 64.96 ± 11.33 years. Most of the patients were males (52.4%). The majority of the patients had comorbid disease conditions (86.7%). The time since the diagnosis of HF was 8.43 ± 4.46 years. Approximately half of the patients were hospitalized due to HF within the previous year (47%), and 35.9% of them were hospitalized two or more times. The majority of patients with HF were diagnosed with NYHA class II or III (38% and 37.3%, respectively), as shown in Table 1.

**Figure 2:**
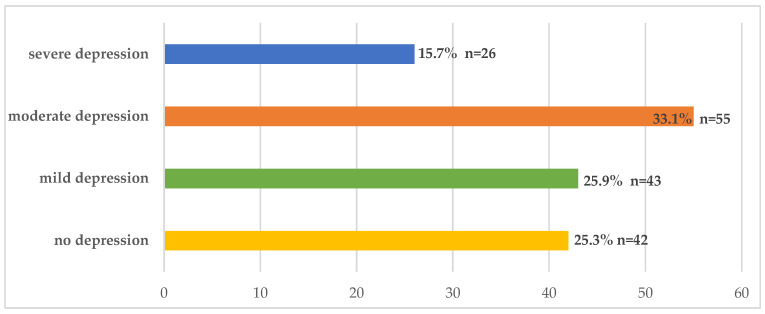
Evaluation of depression levels based on BDI scores among the study participants.

Figure 2 illustrates the assessment of depression levels based on BDI scores among the study participants. Nearly half of the participants had moderate or severe depression (33.1% and 15.7%, respectively). The mean MLHFQ score of the study participants was 54.15 ± 18.20, which indicates a relatively poor HRQOL. Table 2 presents the associations of depression and HRQOL among patients with HF based on the use of the BDI and MLHFQ, respectively. As the depression levels of the patients increased, the HRQOL decreased markedly. Patients without depression had the highest HRQOL (36.52 ± 15.66), whereas patients suffering from severe depression had the lowest HRQOL (71.46 ± 12.4).

Table 3 shows the depression level and HRQOL of patients with HF using the BDI and MLHFQ, respectively. There were significant findings in terms of depression and HRQOL related to poor education level, an HF diagnosis of more than 3 years, a number of HF hospitalizations, polypharmacy, and NYHA class IV. On the other hand, when the participants’ BDI total scores were stratified in consideration of demographic characteristics, neither the age range nor sex was significantly different.

Table 4 shows associations of the BDI and MLHFQ scores with the comorbid disease conditions of the study participants. The depression level was high, particularly among patients who were suffering from arrhythmias (30.44 ± 7.97), whereas HRQOL was remarkably low in patients who were suffering from COPD (66.93 ± 14.99). With respect to the associations of BDI and MLHFQ scores with the number of medications used by the study participants, the depression level was particularly high among patients who were on polypharmacy with 18 medicines (40). On the other hand, the HRQOL was reported to be low among patients who were on polypharmacy with 10 medicines (70 ± 12.73), as shown in Table 5.

Table 6 presents the findings of the multiple linear regression analysis evaluating the effects of age, duration of HF diagnosis, number of comorbid diseases, and number of drugs used on the BDI. There was an insignificant relationship between age and BDI score (0 < r: −0.14 ≤ 0.30), and significant relationships between duration of HF diagnosis, number of comorbid diseases, number of drugs used, and BDI score [(0.30 < r: 0.31/0.43/0.43 ≤ 0.70), respectively]. In other words, age does not play a substantial role in explaining depression levels; in contrast, the duration of HF diagnosis, number of comorbid diseases, and number of drugs used were identified as significant predictors of depression levels. Moreover, relationships of all the independent variables with the BDI are evaluated with multiple correlation coefficients; there is a moderately significant relationship (0.30 < R: 0.53 ≤ 0.70). The multiple regression model yielded significant results, and all the independent variables explained 26% of the BDI variance (F: 15.19; *p*: 0.00 < 0.001; R^2^: 0.28). In addition, when the standardized beta coefficients (β) were assessed, the number of comorbid diseases (β: 0.27; t: 3.30), the number of drugs used (β: 0.22; t: 2.70), and the duration of HF diagnosis (β: 0.20; t: 2.84) were significantly associated with BDI variance. In addition, the findings of the simple linear regression analysis show the explanatory power of the BDI on the MLHFQ. The BDI has positive and significant explanatory power for the MLHFQ (F: 168.29; R2: 0.51; t: 12.97; *p* < 0.001), and 51% of the change in the MLHFQ score is explained by the BDI (R2: 0.51).

## 4. Discussion

Compared with other chronic illnesses, comorbid depression is a more prevalent problem in patients with HF. This leads to significant reductions in HRQOL and is associated with elevated rates of morbidity and mortality, particularly in the elderly population [35,36]. The findings of the present study showed that nearly half of the outpatients with HF had moderate-to-severe depression on the basis of their BDI scores. These findings are in accordance with those of the previous studies reporting high levels of anxiety and depression among Turkish patients with HF [37,38]. Moreover, the mean MLHFQ score of the study participants was poor, indicating a low HRQOL. Similarly, these findings are in agreement with those of the study conducted by Metin and Helvacı [38], which reported poor quality of life among patients with HF in Ankara city, Türkiye, using the World Health Organization Quality of Life Short Form. Another study by Demir and Unsar [39] in Edirne City, Türkiye, reported similar findings with low quality of life among patients with HF using the left ventricular dysfunction (LVD-36) (quality of life) scale.

Several factors affect the level of depression and HRQOL, such as age, sex, educational level, marital status, and the presence of other diseases. The findings concerning the factors influencing depression severity and patients’ HRQOL were particularly noteworthy. The factors associated with high levels of depression and poor HRQOL were older age, low educational level, unemployment, multiple hospitalizations, and NYHA HF stages III and IV.

With respect to age, as the population ages, the prevalence of HF is predicted to increase among those over 65 years [40]. In this study, the mean age of the study participants was 64.96 ± 11.33 years. These findings are consistent with previous studies showing that the prevalence of HF increases with age [26,40].

The nature of HF disease makes assessing depression and HRQOL one of the most critical responsibilities. Long-term treatment plans, emotional instability, and significant lifestyle modifications are characteristics of HF as a chronic condition. Moreover, as the symptoms of HF worsen, patients experience a steady progression of the disease and a decline in their physical capacity [41,42,43]. A study by Mulugeta et al. [27] conducted at the cardiac outpatient clinics in Addis Ababa, Ethiopia, reported consistent findings. The study revealed significant associations between depression and comorbid diabetes mellitus, NYHA class IV, and patients’ intake of more than five medicines.

In the present study, participants who were primary or high school graduates had significantly greater depression levels and lower HRQOL than university graduates did. Job status was another factor that was linked to the present findings, and a significant association was identified between being unemployed and the level of depression and HRQOL. Compared with employees, unemployed patients experienced worse HRQOL and higher levels of depression. This may be because a favorable economic status is linked to a sense of security and socialization, which has an important effect on patients’ emotional status and HRQOL. Another statistically significant factor that contributes to high depression and poor HRQOL is the severity of the disease. In particular, patients with NYHA class IV disease had significantly greater depression levels and lower HRQOL than those with lower NYHA class. The finding can be expected since patients with a higher disease stage of HF experience more severe and significant symptoms. Notably, patients feel more uncomfortable and less independent, which predisposes them to greater deterioration in HRQOL. Similarly to our findings, relevant studies also reported a high prevalence of depression in patients with severe HF and a poor HRQOL [27,44,45,46]. For example, Pena et al. [47] reported that patients with NYHA grade IV were more depressed than those with NYHA grades II or III.

Among other findings in the present study is the association between the rate of hospitalization and the level of depression and HRQOL. There is a reciprocal relationship between deterioration of health status in patients with HF and negative emotions and poor HRQOL. In particular, this fact is also linked with the severity of HF, in which patients with severe disease or higher NYHA class usually have higher rates of hospital admissions. Patients who are hospitalized many times are at risk of acute exacerbations with respect to their health status, which further explains the higher level of depression and poor HRQOL [43,48]. In the present study, compared with patients who were never or only hospitalized once, patients who had been hospitalized multiple times in the previous year had higher levels of depression and lower HRQOL. These findings are in accordance with other studies by Aggelopoulou et al. [26], Mulugeta et al. [27], Chu et al. [44], Ventoulis et al. [45], and Liu et al. [46]. Aggelopoulou et al. [26] conducted an observational study in Greece to assess the levels of anxiety, depression, and quality of life among patients with HF.

In the present study, there was no statistically significant difference between men and women in terms of depression levels or HRQOL. This might be related to the small sample of participants enrolled in the present study. This finding was consistent with those of Aggelopoulou et al. [26] and Mulugeta et al. [27], but contrasts with the results of other studies. Women had an impaired emotional state compared with men in the study by Chu et al. [44], and women had a higher level of depression than men in the study by Ramos et al. [49].

Our study revealed that depression levels were high, particularly among patients who were suffering from comorbidities, such as arrhythmias, neurological diseases, and COPD. Similarly, HRQOL was remarkably low in patients who were suffering from COPD, kidney failure, arrhythmias, and malignant diseases. Among HF patients with comorbidities, the economic burden of complications, inadequate disease management, and generally poor health status can be debilitating and stressful. Feelings of frustration and anxiety may result from this, further exacerbating depression and lowering HRQOL in this group of people [50,51,52]. Similar findings have been reported in other studies [27,53,54]. However, a study conducted by Çavuşoğlu et al. [55] revealed that the most prevalent comorbidities among HF patients in Türkiye were hypertension, atherosclerotic cardiovascular disease, dyslipidemia, diabetes mellitus, COPD, anemia, and atrial fibrillation.

Moreover, our study revealed that there is a significant relationship between the number of comorbid diseases and depression level. The depression level was high, particularly among patients who were suffering from comorbidities, such as arrhythmias, neurological diseases, and COPD. Similarly, HRQOL was remarkably low in patients who were suffering from COPD, kidney failure, arrhythmias, and malignant diseases. On the other hand, our findings also reported an association between the number of medications taken daily and the level of depression and HRQOL, as it was observed that 51% of the change in MLHFQ scores is explained by BDI.

To lessen the impact of polypharmacy on mental health outcomes in patients with HF, it is crucial to closely monitor patients’ medication regimens and educate them about medication management, including the importance of adherence and the possible occurrence of side effects. Our findings also revealed an association between the number of medications taken daily and the level of depression and HRQOL. This could be related to the increased frequency of adverse drug reactions, which can lead to negative emotions, including depression [56,57]. This finding is consistent with a systematic review and meta-analysis that indicated that polypharmacy was substantially linked to a higher incidence of depression in HF adults [56].

### 4.1. Recommendations

In light of the data in our study and to the best of our knowledge, this is the first study that provides an important comprehensive contribution to comorbid depression, and consequently HRQOL, taking into consideration the diverse factors of participants in association with comorbid depression and HRQOL among outpatients with HF in Bursa Province, Türkiye. Given these findings, the following is recommended for Turkish cardiology healthcare professionals who provide care services for patients with HF:

- Identifying psychological comorbidities in HF outpatient care is a crucial first step in providing this population with specialized, individualized care.
- Given the significant incidence of depression and the resulting low HRQOL, incorporating regular assessments of clinical and sociodemographic factors for depression symptoms into standard care procedures is critical.
- Closely monitor patients’ health behaviors, particularly those who are elderly and have low levels of education.
- Plan necessary interventions and follow up on patients’ psychosocial well-being on a regular basis to improve their quality of life.
- Create training programs specific to patient characteristics regarding the significance of self-care and health behaviors in the management of the disease.
- Consider comorbid conditions that are prevalent in the Turkish HF population. Importantly, prompt and effective management of these comorbidities may enhance the clinical results and reduce the disease burden.

### 4.2. Limitations

This study has several limitations that should be considered. This was a descriptive cross-sectional study that relied on self-reported data to assess depression and other independent variables, which may be prone to recall bias and social desirability bias. Second, patients with HF were included from a single center, which was not large enough and may not be fully representative of the broader target population and its potential impact on the generalizability of the findings. Third, depression symptoms can change over time, and this study collected data from only one time point. Finally, the impact of medication adherence and the management of depression and anxiety on clinical outcomes was beyond the scope of this study. Therefore, given these limitations into consideration, further cohort studies, including longitudinal and interventional studies with larger samples, should be conducted to gain further insights into the associations between variables and monitor changes in depression symptoms over time in patients with HF.

## 5. Conclusions

The results of this study revealed that comorbid depression and HRQOL are closely related. This was observed in nearly half of the patients with HF who had comorbid moderate and severe depression, which is associated with poor HRQOL. The factors associated with high depression and poor HRQOL were a low education level, a duration of HF diagnosis of more than 3 years, an increased number of HF hospitalizations, polypharmacy, comorbid disease conditions, and a NYHA class IV diagnosis. Given these findings, identifying psychological comorbidities in HF outpatient care is a crucial first step in providing this population with specialized, individualized care. Given the significant incidence of depression and the resulting low HRQOL, it is critical that healthcare providers incorporate regular assessments of clinical and sociodemographic factors for depressed symptoms into their standard care procedures.

## Figures and Tables

**Table 1:** Demographic characteristics of the study participants (N = 166).

Variables		Number (N)	Percentage (%)
Sex	Female	79	47.6
	Male	87	52.4
Age (years)	≤49	12	7.2
	50–59	34	20.5
	60–69	63	38
	70–79	42	25.3
	80–89	15	9
Marital status	Single	2	1.2
	Married	118	71.1
	Divorced	13	7.8
	Widowed	33	19.9
Education level	Literate	18	10.8
	Primary school graduate	56	33.7
	Secondary school graduate	22	13.3
	High school graduate	46	27.7
	University graduate	24	14.5
Working status	Working	63	38
	Retired	64	38.6
	Unemployed/Householders	39	23.4
Cigarette smoking	I do not smoke at all	56	33.7
	I used to smoke, but not anymore	68	41
	I smoke regularly	17	10.2
	I smoke occasionally	25	15.1
Presence of comorbid disease	Yes	144	86.7
	No	22	13.3
Presence of polypharmacy	Yes	122	73.5
	No	44	26.5
Hospitalization due to heart failure within the last year	Yes	78	47
	No	88	53
Number of hospitalizations due to heart failure within the last year	1	50	64.1
	2 or more	28	35.9
NYHA classification	Class I	24	14.5
	Class II	63	38
	Class III	62	37.3
	Class IV	17	10.2
Duration of heart failure diagnosis (months mean ± SD)			53.52 ± 9.34

SD: Standard deviation, N: number of participants.

**Table 2:** Association of BDI and MLHFQ total scores.

Depression Level	BDI Total Score	MLHFQ Total Score (Mean ± SD)
No depression	0–13 points	36.52 ± 15.66
Mild depression	14–19 points	50.72 ± 10.64
Moderate depression	20–28 points	62.11 ± 14.59
Severe depression	29–63 points	71.46 ± 12.4

BDI: Beck Depression Inventory, MLHFQ: Minnesota Living with Heart Failure Questionnaire, SD: standard deviation.

**Table 3:** BDI and MLHFQ score distributions of participants according to demographic characteristics.

Variables		BDI Total Score (Mean ± SD)	*p*-Value	F	MLHFQ Total Score (Mean ± SD)	*p*-Value	F
Age (years)	≤49	21.00 ± 9.36	0.39	1.03	55.75 ± 15.52	0.10	1.96
	50–59	22.00 ± 6.86			59.53 ± 20.03		
	60–69	19.94 ± 9.87			54.29 ± 18.13		
	70–79	20.38 ± 10.07			52.64 ± 17.39		
	80–89	16.40 ± 6.47			44.33 ± 16.36		
Marital status	Single	8.50 ± 2.12	0.001	8.60	24.50 ± 0.71	0.001	4.66
	Married	18.58 ± 7.44			52.12 ± 17.38		
	Divorced	21.23 ± 13.31			57.54 ± 21.27		
	Widowed	26.42 ± 9.93			61.88 ± 17.54		
Education level	Primary school graduate	20.45 ± 8.83	0.001	7.39	57.84 ± 17.13	0.02	3.10
	Secondary school graduate	20.95 ± 8.53			55.86 ± 18.41		
	High school graduate	18.39 ± 8.46			49.67 ± 16.59		
	University graduate	15.79 ± 6.37			47.04 ± 16.55		
Working status	Working	19.32 ± 10.65	0.01	4.42	54.05 ± 20.38	0.47	0.76
	Retired	18.88 ± 7.30			52.48 ± 18.55		
	Unemployed/Householders	23.92 ± 8.19			57.05 ± 13.61		
Cigarette smoking	I do not smoke at all	22.70 ± 8.34	0.01	4.30	54.66 ± 18.41	0.04	2.83
	I used to smoke, but not anymore	20.63 ± 9.10			57.74 ± 19.15		
	I smoke regularly	16.24 ± 10.43			46.00 ± 13.87		
	I smoke occasionally	16.32 ± 7.98			48.80 ± 15.76		
Duration of heart failure diagnosis	<1 year	18.67 ± 7.63	0.001	5.16	53.62 ± 17.99	0.001	3.53
	≤1 year–<2 years	16.14 ± 8.16			43.76 ± 22.07		
	≤2 years–<3 years	14.56 ± 5.62			46.41 ± 19.21		
	≤3 years–<4 years	21.36 ± 8.35			55.54 ± 12.16		
	≤4 years–≤5 years	24.81 ± 8.16			58.81 ± 13.09		
	<5 years–≤10 years	22.24 ± 11.19			58.88 ± 18.58		
	10 years <	23.20 ± 10.63			62.65 ± 20.36		
Number of hospitalizations due to heart failure within the last year	Never	16.06 ± 6.61	0.001	29.01	47.16 ± 15.89	0.001	23.84
	Once	23.42 ± 8.09			57.18 ± 17.21		
	Twice or more	27.64 ± 10.63			70.71 ± 14.95		
NYHA Classification	Class I	11.67 ± 4.10	0.001	43.32	36.25 ± 14.42	0.001	36.65
	Class II	16.02 ± 5.36			46.37 ± 14.63		
	Class III	25.03 ± 8.02			65.90 ± 12.78		
	Class IV	30.41 ± 9.61			65.41 ± 17.19		
**Variables**		**BDI Total Score** **(Mean ± SD)**	***p***	**t**	**MLHFQ Total Score (Mean ± SD)**	***p***	**t**
Sex	Female	21.49 ± 8.93	0.09	1.72	55.18 ± 16.87	0.49	0.69
	Male	19.08 ± 9.13			53.22 ± 19.48		
Presence of comorbid disease	Yes	20.38 ± 8.35	0.69	0.40	54.17 ± 17.65	0.97	0.04
	No	19.23 ± 13.18			54.00 ± 22.28		
Presence of polypharmacy	Yes	21.56 ± 9.18	0.01	3.22	54.76 ± 17.00	0.52	0.64
	No	16.55 ± 7.83			52.45 ± 21.49		
Hospitalization due to heart failure within the last year	Yes	24.94 ± 9.24	0.001	7.04	62.04 ± 17.60	0.001	5.72
	No	16.06 ± 6.61			47.16 ± 15.89		

SD: Standard deviation, significant at *p*-value < 0.05.

**Table 4:** Association between BDI and MLHFQ scores with the comorbid disease conditions of the study participants.

Comorbid Diseases	BDI Total Scores (Mean ± SD)	MLHFQ Total Score (Mean ± SD)
Hypertension	21.91 ± 8.93	55.02 ± 17.66
Diabetes	22.4 ± 9.05	58.52 ± 17.76
Dyslipidemia	22.88 ± 9.84	56 ± 14.48
Hypothyroidism	20.84 ± 8	52.79 ± 13.52
Coronary Artery Disease	24.86 ± 11.81	58.14 ± 15.66
COPD	25.14 ± 11.32	66.93 ± 14.99
Kidney Failure	24.69 ± 7.47	65.62 ± 16.59
Respiratory Diseases	21.17 ± 5.32	55.50 ± 19.34
Neurological Diseases	25.45 ± 10.81	58.91 ± 21.03
Arrhytmias	30.44 ± 7.97	63.67 ± 14.07
Benign Prostatic Hyperplasia	19.67 ± 5.29	55.78 ± 9.77
Gastrointestinal Diseases	11.86 ± 3.98	40.29 ± 10.21
Anemia	22.33 ± 10.54	53.83 ± 17.67
Malignant Diseases	24.80 ± 6.06	63.00 ± 15.02

SD: Standard deviation.

**Table 5:** Association between BDI and MLHFQ scores with the number of medications used by the study participants.

Number of Medications	BDI Total Scores (Mean ± SD)	MLHFQ Total Score (Mean ± SD)
1	7	45
2	17.4 ± 6.5	47.6 ± 19.63
3	15.5 ± 5.91	47.4 ± 26.7
4	17.11 ± 8.69	55.39 ± 20.39
5	17.83 ± 9.26	45.79 ± 14.73
6	19.18 ± 6.9	53.21 ± 18.33
7	19.67 ± 5.58	52.24 ± 10.87
8	22 ± 11.17	54.55 ± 21.8
9	25.91 ± 8.79	62.45 ± 11.67
10	32.43 ± 8.32	70 ± 12.73
11	28.14 ± 8.34	68.43 ± 8.72
12	20.5 ± 9.19	64.5 ± 27.58
13	22	59
18	40	60

SD: Standard deviation.

**Table 6:** Regression analysis results regarding the prediction of participants’ depression levels.

Independent Variables	B	SH	β	t	*p*-Value	r (Binary)	Tolerance	VIF
Age	−0.03	0.05	−0.04	−0.59	0.56	−0.14	0.97	1.04
Duration of heart failure diagnosis	0.39	0.14	0.20	2.84	0.01	0.31	0.92	1.09
Number of comorbid diseases	1.86	0.56	0.27	3.30	0.001	0.43	0.70	1.44
Number of drugs used	0.81	0.30	0.22	2.70	0.01	0.43	0.67	1.50

R: 0.53 R^2^: 0.28 F: 15.19 *p*: 0.001.

## Data Availability

The data presented in this study are available on request from the corresponding author, as the data are not publicly available due to privacy or ethical restrictions.

## Abbreviations

The following abbreviations are used in the manuscript:

ACC/AHA	American College of Cardiology/American Heart Association
BDI	Beck Depression Inventory
COPD	Chronic Obstructive Pulmonary Disease
CV	Cardiovascular
ESC	European Society of Cardiology
HF	Heart failure
HFSA	Heart Failure Society of America
HRQOL	Health-related quality of life
LVD-36	Left ventricular dysfunction (quality of life) scale
MLHFQ	Minnesota Living with Heart Failure Questionnaire
NYHA	New York Heart Association
